# A cautionary note on the potential pitfalls of using N-terminal truncated CD63 to label small extracellular vesicles

**DOI:** 10.1038/s41598-025-91597-6

**Published:** 2025-03-01

**Authors:** Elias Sulaiman, Derek M. Yellon, Sean M. Davidson

**Affiliations:** The Hatter Cardiovascular Institute, University College London, 67 Chenies Mews, London, WC1E 6HX UK

**Keywords:** Nanoparticles, Multivesicular bodies

## Abstract

Small extracellular vesicles (sEV) are nanosized vesicles that facilitate intracellular communication. A significant research obstacle is the isolation of sEV devoid of non-sEV contaminants. Immunoaffinity capture with sEV-specific antibodies is an attractive approach to purifying sEV, but it risks disrupting the vesicles during antibody dissociation. Furthermore, immunoaffinity capture may require the modification of EV-specific proteins for the incorporation of tags on the EV surface, with unknown implications on EV production and function. The aim of this study was to investigate whether a previously reported CD63 truncation is efficient for the incorporation of small tags on the extravesicular surface. We therefore conjugated ALFA-tag to N-terminal-truncated CD63, and included nanoluciferase at the C-terminus, for luminescent tracing of the sEV. Full-length CD63-nanoluciferase was used as a control. Plasmid constructs expressing these proteins were transfected into HEK293 cells. In contrast to a previous report, the N-terminal truncation of CD63 impaired its membrane localisation and reduced the yield of EVs. Further investigation revealed that some of the tagged CD63 was co-localized with aggresomes and was preferentially secreted from the cells as soluble protein rather than being associated with sEV. These results demonstrate that CD63 truncation can impair its function and EV yield, potentially generating misleading results.

**Supplementary Information:**

The online version contains supplementary material available at 10.1038/s41598-025-91597-6.

## Introduction

Extracellular vesicles (EVs) are a diverse group of nanosized vesicles which facilitate intracellular communication. They are comprised of a lipid bilayer membrane and carry a variety of molecules, such as proteins and nucleic acids, which serve as their functional cargo^1,2^. EVs can be divided into two major subtypes depending on their biogenesis: ectosomes, which are shed from the plasma membrane and are further subcategorised into microvesicles, apoptotic bodies and oncosomes; and exosomes, which are generated via an endocytic pathway involving fusion of multivesicular bodies (MVBs) with the plasma membrane^3^. Although these EV subtypes usually overlap in size, guidelines encourage the use of the term “small extracellular vesicles” (sEVs) to include all EVs with a diameter of < 200 nm^4^.

One of the biggest challenges in the field of EV research, is to isolate EVs devoid of contamination from non-EV proteins and lipoproteins. It is also challenging to separate different EV subtypes such as exosomes, from other EVs, due to the size overlap and the expression of some surface markers. The most commonly used methods for EV concentration and purification from biological fluids, either alone or combined, are ultracentrifugation, ultrafiltration, size exclusion chromatography (SEC) and immunoaffinity isolation. Ultracentrifugation has been characterised as the gold standard technique for concentrating EVs^5–7^, although more recently, combinations of methods such as ultrafiltration (e.g., tangential flow filtration) followed by SEC are being utilised more frequently, as they can lead to more pure vesicle isolations, are less time consuming and do not require expensive instrumentation. Immunoaffinity capture is an attractive approach, since antibodies against surface markers of EVs—most commonly CD63, but also CD9 and CD81—can potentially be used to isolate solely those vesicles that carry the “EV-specific” markers of interest, with minimal co-isolation of contaminants. On the other hand, a caveat with the use of immunoaffinity is that if the purified EVs are intended for functional studies, the act of dissociating them from the antibody can cause physical damage or disruption to the vesicles.

The tetraspanin CD63 is often described as an “EV-specific” marker, as it can be found on the surface of various EVs and has been widely utilised in engineering studies for EV tracking or cell targeting purposes. For example, several studies have fused a small luciferase sequence termed ‘Nluc’ to the intravesicular C-terminus of CD63, which allows the tracking of EV cellular uptake and in vivo biodistribution in animal models. However, as both the N- and C-termini of CD63 are intravesicular, an engineered epitope to be exposed to the outer surface of the EVs, must be inserted within one of the extracellular loops, which may potentially affect CD63 localisation and/or vesicle biogenesis. Indeed, the large extracellular loop is essential for correct incorporation of the CD63 protein into EVs^8^. Recently, however, Curley et al., claimed that removal of the first 46 amino acids (consisting of the N-terminus, the first transmembrane domain and part of the first extracellular loop) does not affect the proper localisation of CD63 on the vesicular surface, while making the N-terminal extravesicular and available for conjugation to peptides of interest^9^. Therefore, in the present study, we used an N-terminal truncated CD63 and conjugated a low-affinity peptide tag called ALFA-tag (SRLEEELRRRLTE) on the novel extravesicular N-terminus. This was performed with the hope to develop a new approach to isolate EVs using a readily reversible immunoaffinity approach, as the tag on the outer surface of EVs could enable their purification by binding with the specific anti-ALFA nanobody to facilitate purification^10^. We also included a nanoluciferase sequence (Nluc) at the (intravesicular) C-terminus to allow them to be luminescently traced.

## Materials and methods

### Plasmid DNA design and bacterial extraction

The plasmid DNA pDB30, obtained from the Fussenegger lab^11^, encodes human CD63 with a Nanoluciferase sequence tethered to the C-terminus of the tetraspanin, dubbed ‘CD63-Nluc’. This plasmid was used as a scaffold to prepare a new plasmid DNA, which encodes a truncated form of CD63 named ‘ALFA-tCD63-Nluc’. The new protein has its first 46 amino acids removed, thus creating a new N-terminus, which will be exposed to the outer surface of the EV once incorporated on their membrane, and a small peptide named ‘ALFA’ was added to the beginning of the sequence. In order to investigate the effect of the ALFA tag in CD63, we also created another plasmid DNA termed ‘CD63-Nluc-ALFA’, where CD63 retains its full form and the ALFA tag is tethered to the N-terminus, which results in the intravesicular space.

For the synthesis of the ALFA-tCD63-Nluc plasmid DNA, a double stranded cDNA was designed by us and synthesized by IDT (Integrated DNA Technologies, Belgium), where the first 138 nucleotides of the CD63 gene were removed, and the ALFA tag was added. Both the new insert and the pDB30 vector were cut using the HindIII and EcoRI restriction sites, and the insert was ligated with the dephosphorylated vector creating ALFA-tCD63-Nluc.

For the synthesis of the CD63-Nluc-ALFA plasmid DNA, pDB30 was cut using the KfII and XbaI restriction sites and was run on low-melting point agarose gel. The band containing the nucleic acid was extracted, and in order to replace for the lost part of Nluc that was removed, a new insert was synthesized by IDT and incorporated in the vector with a T4 DNA ligase as previously mentioned^11^. The newly synthesized plasmid DNA was sequenced by Source Bioscience UK.

All three plasmid DNAs were used to transform *E. coli* JM101 bacteria (Promega, L2005) with the heat shock method as previously described^12^. After their recovery, they were grown on agar plates with ampicillin prior to their plasmid DNA extraction with Qiagen Plasmid Maxi prep kit (Qiagen, Valencia, California, USA).

### Cell culture

HEK293 cells (Human embryonic kidney cells, obtained from American Type Culture Collection, ATTC), were cultured in DMEM (Gibco) supplemented with 10% FBS (Fetal bovine serum, Sigma-Aldrich) and 1% penicillin-streptomycin at 37 °C with 5% CO_2_.

For HEK293 cells to express the modified forms of CD63, overnight transfection was performed at ~ 70– 80% confluency with each of the purified plasmid DNA mixed with PEI (polyethylenimine, Sigma- Aldrich) at a 1:4 ratio, Then, cells were washed with PBS and incubated in serum-free DMEM for 24 h prior to sEV concentration.

### Concentration of small extracellular vesicles (sEV)

For the concentration of sEV, transfected HEK293 cells were grown in FBS-free DMEM for 24 h after their transfection with each plasmid DNA as previously mentioned. On the day of the sEV concentration, cell media was collected and centrifuged for 10 min at 3,000 g at 4 °C to remove cell debris and the supernatant was subsequently ultrafiltered with Vivaspin 100 kDa 20R and Vivaspin 2R concentrator tubes (Sartorius). The concentrated media was diluted with PBS to 500 µl and was then fractionated with SEC (size exclusion chromatography, qEV 35 nm Izon Science), where 20 samples of 500 µl in volume were collected and stored at − 80 °C until further analysis.

### Nanoparticle tracking analysis (NTA)

To measure the concentration and size (in diameter) of sEV, we utilised a Nanosight LM10-HS (Malvern, UK) with a 488 nm violet laser module. The SEC fractions that were collected were diluted with distilled water and a total of 3 videos of 90 s were acquired and the results were extracted with the NTA 3.1 Build 3.1.54 software. The detection threshold was set at 5, camera level at 15 and the syringe speed pump at 20.

### Dissociation-enhanced lanthanide fluorescence immunoassay (DELFIA)

DELFIA was utilised for the detection of sEV-specific markers as previously described^1,13,14^. High-binding 96-well plates (DY990, R&D Systems) were used for the overnight incubation at 4 °C of 50 µl of the most particle-rich SEC fraction from each isolation, as determined by NTA. After washing the wells with DELFIA wash buffer, 1% BSA in PBS was added for 1 h at room temperature (RT), followed by the addition of 1 µg/ml of CD9, CD63 and CD81 primary antibodies (555370, 556019 and 555675 respectively, BD Biosciences, USA) for 2 h at RT. Next, the secondary biotinylated antibody (Abcam, ab98691) was added to the loaded wells at a concentration of 0.25 µg/ml for 1 h at RT. Lastly, the streptavidin-europium conjugate was diluted in assay buffer and added to the plate (PerkinElmer, 1244-106 and 1244-30) for 1 h at RT, and before the analysis the wells were washed six times with the DELFIA wash buffer before the addition of the DELFIA enhancement buffer (300 rpm for 10 min). The samples were analysed with a PHERAstar plate reader (BMG Labtech) with 337 nm excitation, 620 nm detection, 200 µs integration time and 60 µs lag time.

### Luciferase assay

As previously described, the concentrated sEV are genetically modified to express a small nanoluciferase sequence (Nluc) at the C-terminus of CD63, which allows their accurate tracking in vitro with the Nano-Glo^®^ Dual-Luciferase^®^ reporter assay (Promega). Briefly, either 1 µl of HEK293 cell pellet or 10 µl of each SEC fraction were added to a white 96-well plate with a clear bottom (Greiner Bio-One) with 100 µl of the kit’s buffers, as instructed by the manufacturer, followed by a rigorous shaking for 10 min at 500 rpm. Luminescence readings were recorded with a FLUOstar plate reader (BMG Labtech) in arbitrary units (a.u.)

### Immunostaining of CD63 production in HEK293 cells

Confocal microscopy was utilised to visualise how HEK293 transfection and CD63 truncation changes the expression the modified tetraspanin. HEK293 were cultured on 25 mm coverslips in 6-well plates and transfected with each plasmid DNA as previously described. To prepare cells for immunostaining, they were firstly washed with PBS, fixed for 10 min at 37 °C with paraformaldehyde, permeabilised with 0.1% Triton for 10 min at RT and treated with 5% BSA for 1 h. Subsequently, cells were treated with the CD63 primary antibody diluted in 1% BSA at a 1:100 ratio for 1 h at RT. Then, cells were incubated with the Hoechst 33342 dye to stain the nuclei and the secondary antibody Alexa 488 (ThermoFischer), both at a ratio of 1:2000 diluted in 1% BSA, for 1 h in the dark at RT. Cells were washed three times with PBS before imaging with the Leica TCS SP5 confocal microscope.

For the visualisation of CD63 degradation by aggresomes, we used the commercially available staining kit PROTEOSTAT Aggresome detection kit (Enzo), as this dye fluoresces after binding to misfolded proteins. Transfected and non-transfected HEK293 cells were stained with PROTEOSTAT, in addition to the MG-132-treated cells (positive control) and DMSO-treated (negative control). Quantification of the red and green fluorescence was performed on the confocal images using ImageJ.

### Statistical analysis

Data are plotted as mean ± SEM and generated with GraphPad Prism 9 (GraphPad Software, San Diego, USA). Statistical analysis was performed using one-way ANOVA with Tukey’s post-hoc test with the significant threshold p value was set at < 0.05.

## Results

### Construction and design of the ALFA-tCD63-Nluc EV Tag

**Fig. 1:**
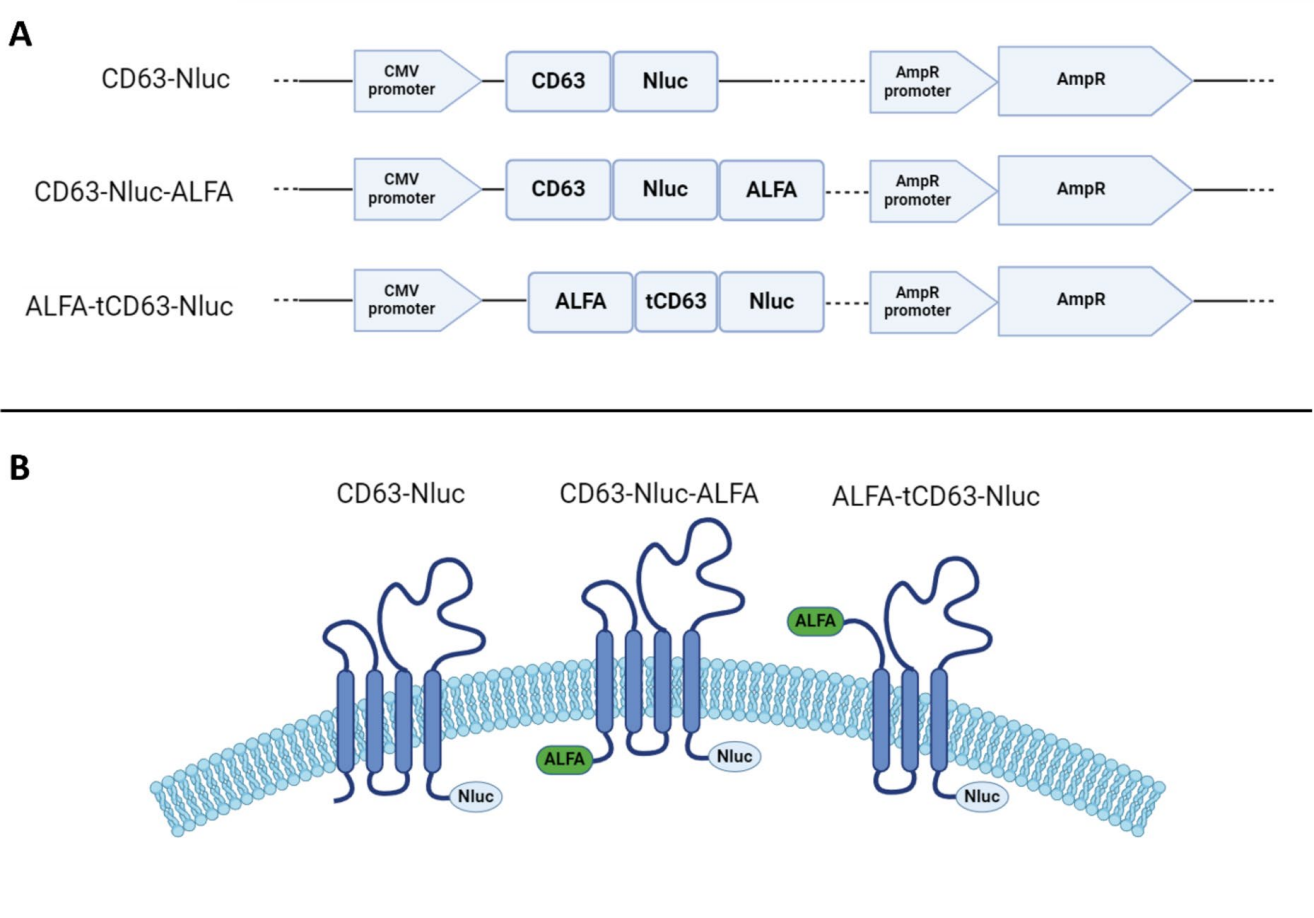
(**A**) Construction of the plasmids used in this study. (**B**) A schematic representation of the expected localisation of the mutant CD63 proteins in the EV membrane shows the exposure of the ALFA tag to outer surface, making it available for nanobody recognition, and the intravesicular location of the small Nluc sequence, which allows the EV carrying these constructs to be luminescent and trackable in vitro. *CMV* cytomegalovirus, *Nluc* nanoluciferase, *AmpR* ampicillin resistance. Images made with http://www.biorender.com.

Curley et al. truncated the N-terminus of CD63 in order to remove the first transmembrane domain and leave a new N-terminus on the outer surface of EV, and found that this resulted in successful CD63 localisation on the vesicular surface, preserving its biological function^9^. In order to confirm this, we removed the same first 46 amino acids of CD63 (tCD63) and attached the small ALFA peptide tag (SRLEEELRRRLTE)^10^ to the new N-terminus, so that we could confirm that the N-terminus was exposed on the outside of the EV by using an antibody to the ALFA tag. The original aim was to use the ALFA tag to purify the labelled EVs, using a low-affinity nanobody to the ALFA tag, that could then be subsequently removed by competition with excess free ALFA peptide^10^. This would allow the recognition of those EV carrying the modified ALFA tag-bearing CD63 marker on their surface from the nanobody and it was hoped would allow us to eventually obtain EVs of greater purity. We used the pDB30 scaffold plasmid DNA, which contains the gene for the expression of CD63-Nluc, to make the new plasmid DNA for the truncated tetraspanin ALFA-tCD63-Nluc and a third plasmid DNA for the expression of the full form of CD63 with the two peptides (CD63-Nluc-ALFA) which is used to examine if the presence of the ALFA tag influences the localisation of the full form of CD63 (Fig. 1). A detailed analysis of the sequences for each plasmid DNA are included in Supplementary Figure S1.

### sEV production in HEK293 is impaired with the expression of ALFA-tCD63-Nluc

**Fig. 2:**
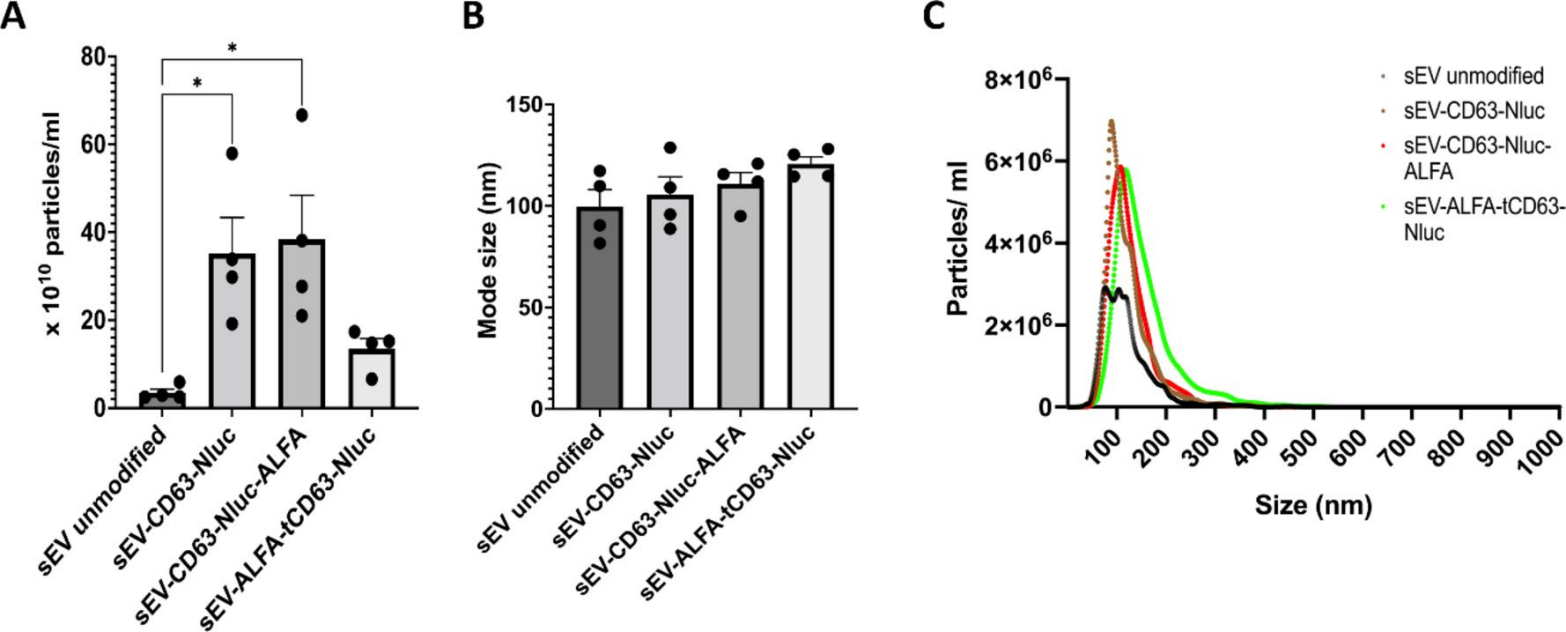
Nanoparticle tracking analysis (NTA) of sEVs isolated from transfected HEK293 cells by ultrafiltration and ultracentrifugation. (**A**) The total number of particles detected by NTA following isolation from HEK293 cells expressing the plasmid constructs indicated. (**B**) The size of the particles assessed by NTA. (**C**) The size distribution of particles detected by NTA. Data shown as mean ± SEM (*n* = 4), *p < 0.05.

sEV were isolated from control and transfected HEK293 cells using ultrafiltration by SEC using a standardised protocol^14^, that leads to single vesicle-enriched SEC fraction (fraction 7 or after elution of 3.0 ml) with the smallest vesicle size of ~ 100 nm mode size (Fig. 2 and Supplementary Fig. S2). NTA revealed that the particle concentration from the non-transfected HEK293 cells (sEV unmodified) was 3.4 × 10^10^ particles/ml, which significantly increased when cells were transfected to express a modified full form of CD63 (35.2 × 10^10^ particles /ml for sEV-CD63-Nluc, and 38.4 × 10^10^ particles /ml for sEV-CD63-Nluc-ALFA). Surprisingly, cells expressing the truncated CD63 with the ALFA tag on the N-terminus (sEV-ALFA-tCD63-Nluc), produced only 13.5 × 10^10^ p/ml (Fig. 2A). No significant differences were observed in the size distribution (Fig. 2C), the mode size (2B) or the mean size (Supplementary Fig. S2B) of sEVs among the groups. These data suggest that, in contrast to the results of Curley et al., in our hands, the truncation of CD63 led to a reduction in sEV production. Importantly, the presence of the ALFA tag is unlikely to have influenced this response, as its presence in the full form of CD63 (i.e., sEV-CD63-Nluc-ALFA) actually significantly increased sEV concentration.

### ALFA-tCD63-Nluc poorly localises on the surface of HEK293-derived sEV

**Fig. 3:**
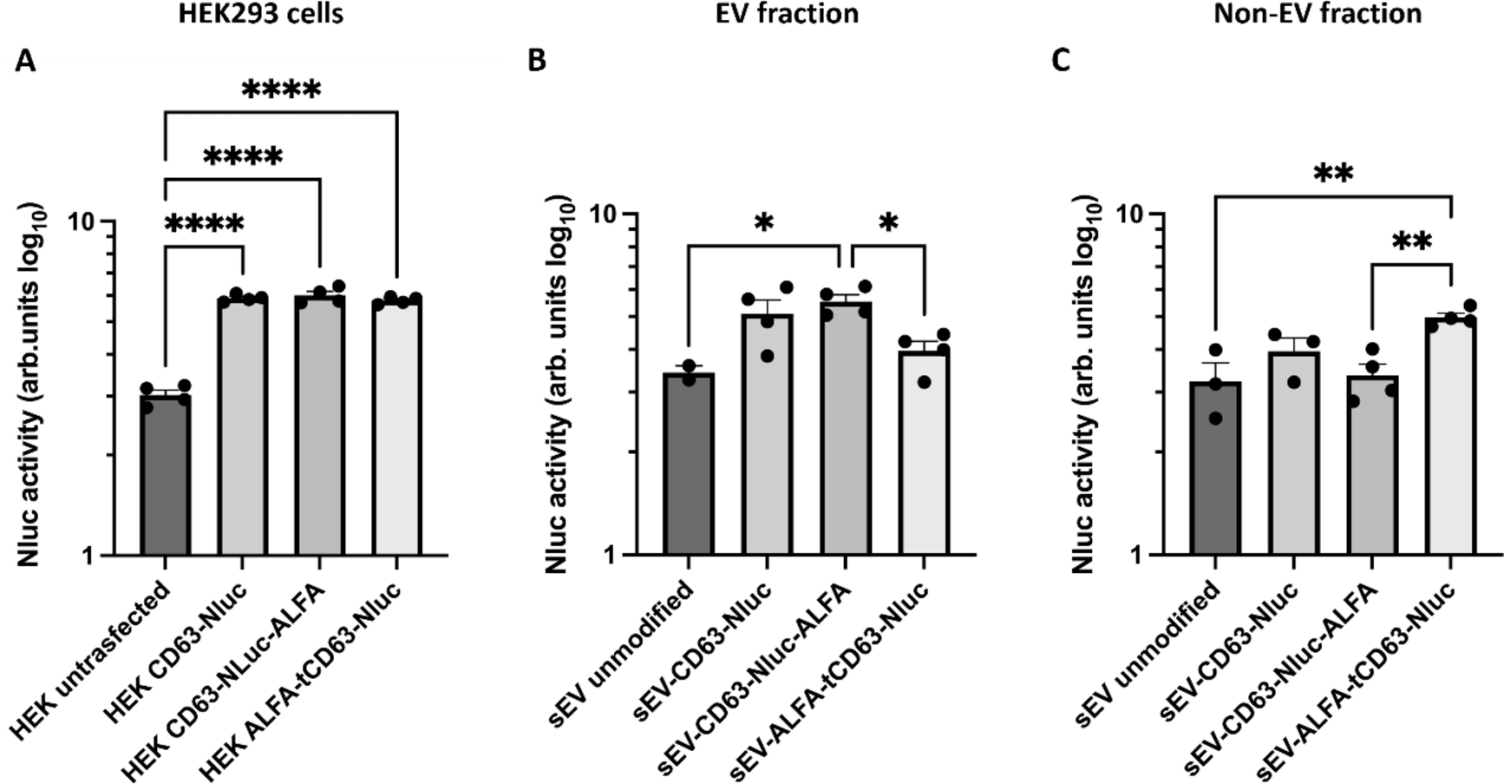
The expression of nanoluciferase (Nluc) in transfected HEK293 cells and the EVs they produce, as measured by luciferase assay. (**A**) Each of the three plasmids led to significantly increased luciferase activity in transfected vs. untransfected HEK293, cells indicating that transfection was successful and all plasmid constructs were expressed. (**B**) sEVs isolated from transfected HEK293 cells exhibited an increase in luciferase as expected, apart from sEV-ALFA-tCD63-Nluc, which suggests that the CD63 protein construct did not insert appropriately into the EV membrane. (**C**) When luciferase activity was measured in the late SEC fractions, which are devoid of vesicles, only the ALFA-tCD63-Nluc construct resulted in significant accumulation of non-vesicular luciferase. Data shown as mean ± SEM (*n* = 4). *p < 0.05, **p < 0.01, ****p < 0.0001. ANOVA performed on log_10_ transformed data.

We performed luciferase assays to investigate if each of the mutant CD63 proteins were expressed to the same degree in the HEK293 cells. Indeed, similar levels of luciferase were measured with each construct in HEK293 cells, indicating they were similarly expressed (Fig. 3A).

Next, we performed luciferase assays to investigate whether the constructs were correctly localized to the EVs. Here, we found that luciferase activity was elevated in EVs from cells expressing CD63-Nluc or CD63-Nluc-ALFA, but not in ALFA-tCD63-Nluc EVs. Unexpectedly, more luciferase was detected in the non-vesicular “late” fraction (free-protein-containing fraction) eluted from the SEC column along with smaller soluble proteins (Fig. 3B, C).

**Fig. 4:**
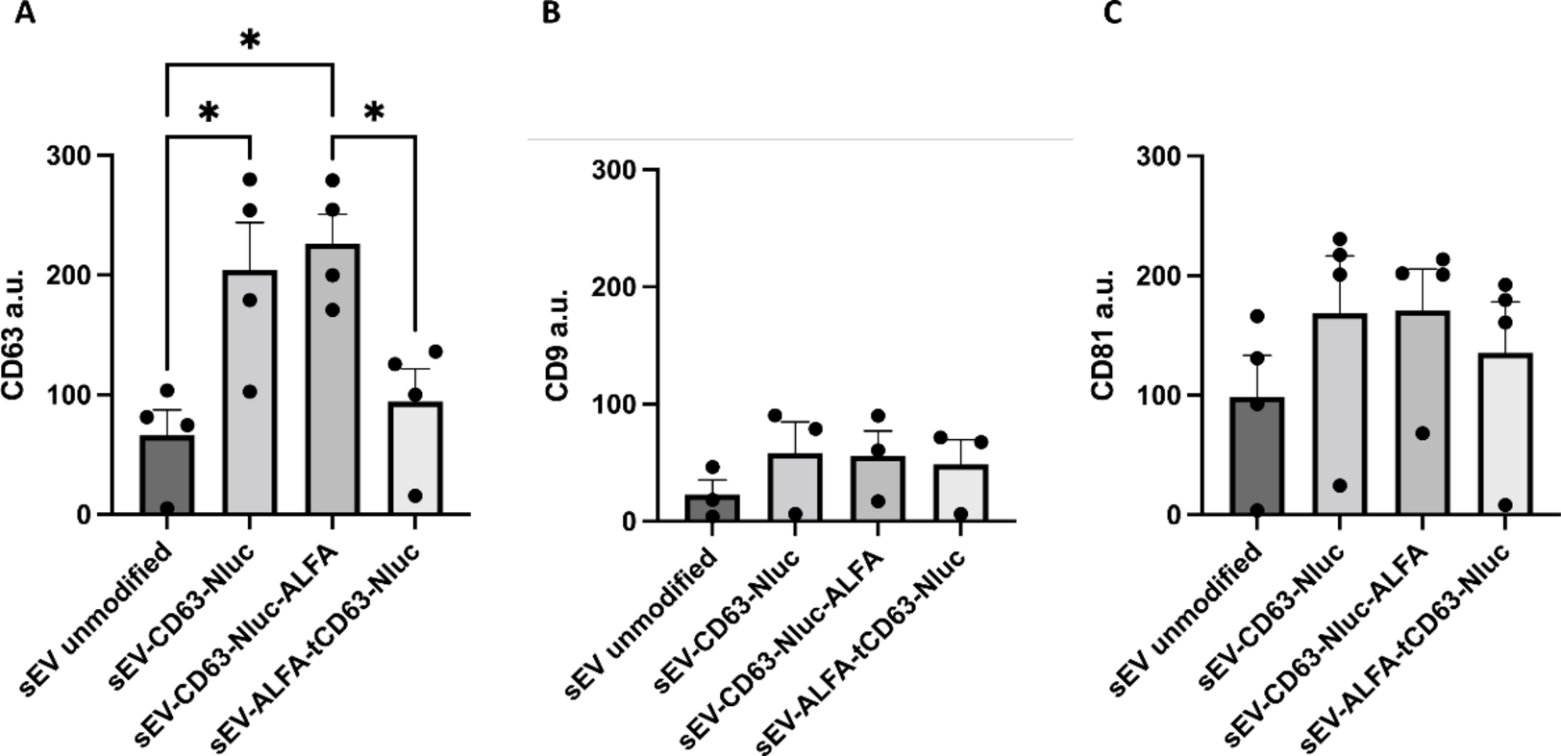
The level of CD63, CD9 and CD81 expression on the surface of the transfected HEK293 sEVs, as measured by antibody labelling and DELFIA. (**A**) CD63-Nluc and CD63-Nluc-ALFA, but not ALFA-tCD63-Nluc, resulted in an increase in surface CD63 expression. Levels of CD9 (**B**) and CD81 (**C**) are similar to all sEV types, indicating that the plasmids did not affect the levels of the other sEVs-specific markers. Data shown as mean ± SEM (*n* = 4), *p < 0.05. *a.u.* arbitrary units of fluorescence.

Following HEK293 transfection to overexpress CD63 in cells, we expected to observe a higher accumulation of the tetraspanin on the surface of EV as previously shown by others^15^. To investigate this, we performed a DELFIA assay (a modified type of ELISA) to measure surface CD63 on sEV. This confirmed that, indeed, overexpression of full-length CD63 exhibited led to increased CD63 on the surface of the sEV produced, and this was independent of the presence of the ALFA tag. In contrast, the ALFA-tCD63-Nluc construct, resulted in significantly lower expression of CD63 on EV, to background levels similar to unmodified sEV (Fig. 4A). We did not observe any changes in other sEV-specific markers CD9 and CD81 (Fig. 4B, C). Taken together, these results indicate that although the novel construct ALFA-tCD63-Nluc is successfully expressed from HEK293, it is unable to properly localise on the surface of sEV.

### ALFA-tCD63-Nluc expression promotes its aggresome degradation

**Fig. 5:**
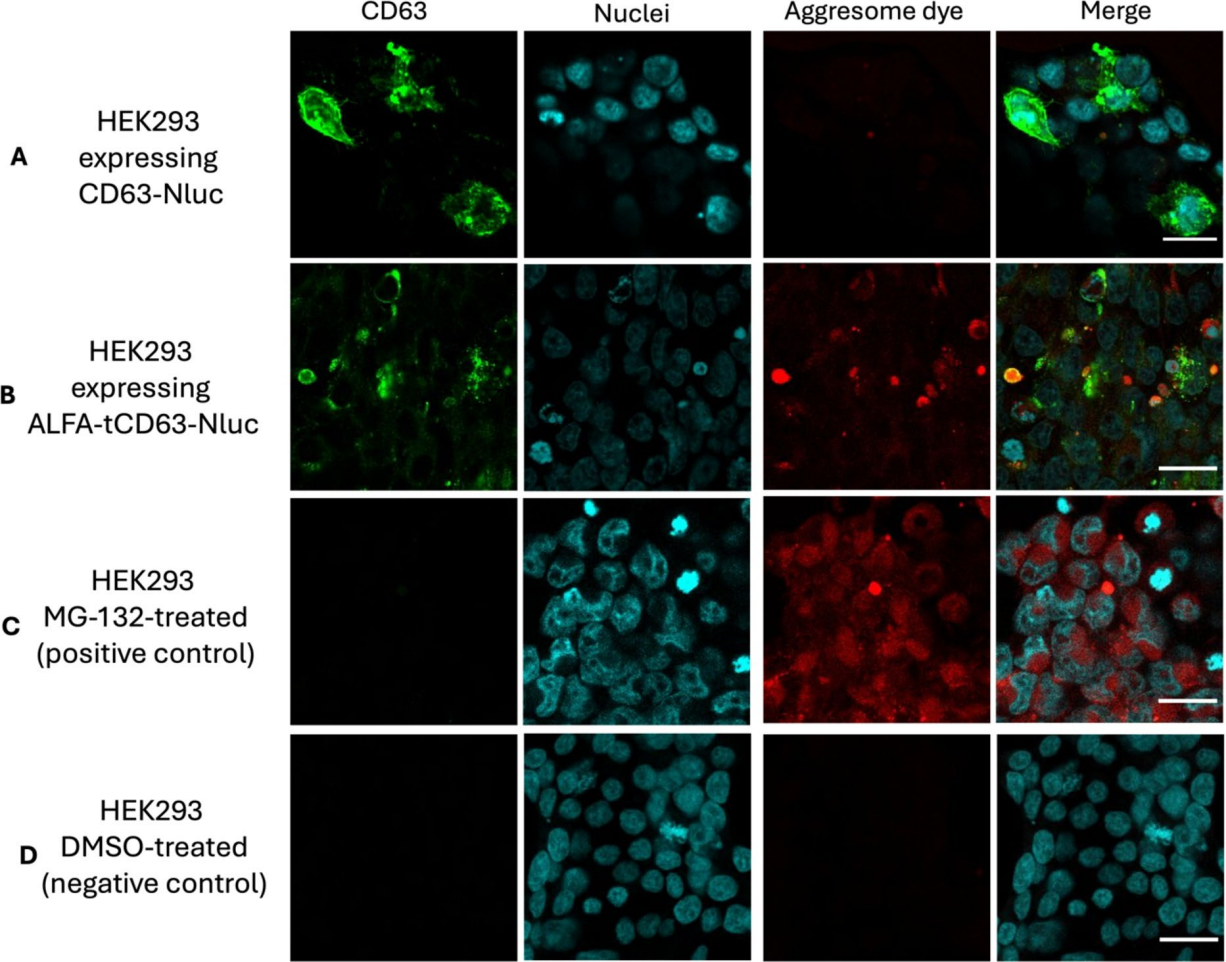
Immunofluorescent staining of aggresomes in transfected HEK293 cells revealed ALFA tCD63-Nluc caused the accumulation of aggresomes. Cells were co-stained for CD63 (green), aggresomes (red) and DNA (blue) 48 h after transfection with the plasmids indicated. CD63 was increased when overexpressed, as expected (**A**), and was uniformly distributed in HEK293 cytosol in the case of CD63-Nluc. However, CD63 expression in cells expressing ALFA-tCD63-Nluc was spherical, green and red fluorescence overlapping, indicating intracellular accumulation of ALFA-tCD63-Nluc within aggresomes (**B**). As a positive control for the formation of aggresomes, HEK293 were treated with the proteasome inhibitor MG-132 (**C**), and DMSO was used as a negative control (**D**). Scale represents 30 μm.

Next, to shed more light on the expression and cellular localisation of the modified CD63, we performed immunostaining of CD63 in transfected HEK293 cells. We also investigated whether the ALFA-tCD63-Nluc construct was being incorporated into aggresomes—inclusion bodies formed by aggregated and misfolded proteins that escape the ubiquitin-proteasome degradation machinery and cleared via autophagy^16–18^. First, we confirmed that the absence of FBS in the culture medium and treatment with the transfection reagent does not influence the expression and cellular localisation of CD63 (Supplementary Fig. S3). Next, we showed that transfection with CD63-Nluc resulted in high green fluorescence intensity (Fig. 5), in agreement with our luminescence experiments in Fig. 3A, which verified the successful overexpression of CD63 in the cells. In contrast, in HEK293 expressing ALFA-tCD63-Nluc, the cells expressing CD63 appeared spherical and were brightly stained with PROTEOSTAT^®^ dye, which fluoresces red when bound to aggresomes (Fig. 5). This indicated that the ALFA-tCD63-Nluc construct indeed forms aggresomes after its cellular expression, thus not allowing the proper incorporation on the vesicular membrane.

**Fig. 6:**
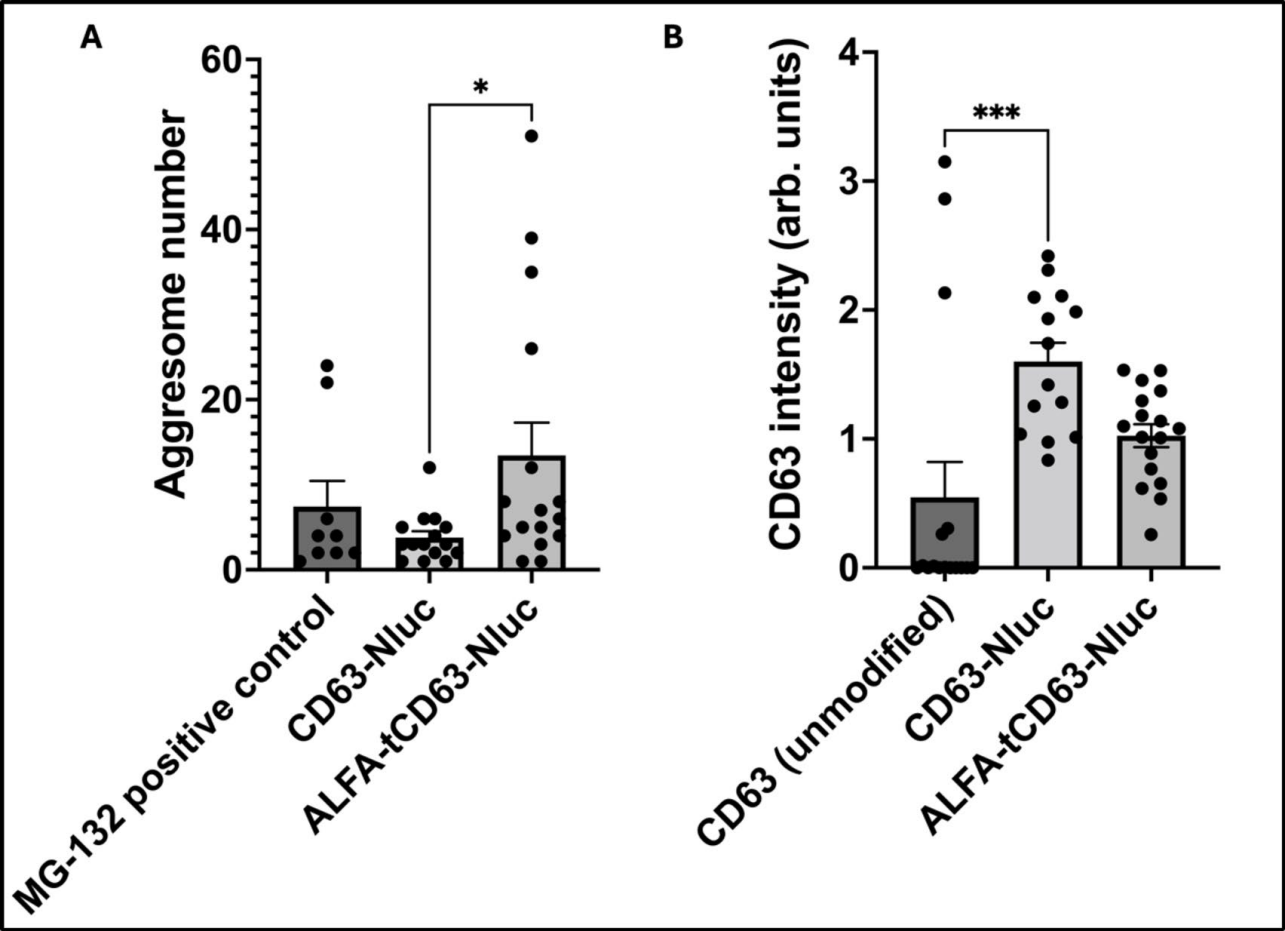
Quantification of CD63 expression and aggresome formation in HEK293 cells from confocal imaging. (**A**) The number of aggresomes formed after the transfection of HEK293 for the expression of ALFA-tCD63-Nluc was significantly higher from the amount spotted for CD63-Nluc-expressing HEK293 cells. (**B**) Quantification of CD63 expression showed significant increase of the tetraspanin in HEK293 expressing CD63-Nluc compared to non-transfected cells, in contrast to HEK293 expressing ALFA-tCD63-Nluc (*p < 0.05). Data shown as mean ± SEM, *p < 0.05 and ***p < 0.001.

Quantification of the confocal microscopy images demonstrated significantly more aggresomes in HEK293 transfected for ALFA-tCD63-Nluc versus CD63-Nluc alone (*p* < 0.05, Fig. 6A), and only higher levels of CD63 (green fluorescence) in the case CD63-Nluc expressing cells (*p* < 0.001, Fig. 6B). We did not find any difference in the size of the aggresomes between the groups (Supplementary Fig. S4).

## Discussion

The challenge of isolating EVs as pure entities from a biological source without the presence of common contaminants, such as proteins and lipoproteins, is one the most important obstacles in the field that must be addressed. One promising approach is immunoaffinity capture, using antibodies highly specific to EVs, which will allow EV purification with minimal disruption to their morphology and biological characteristics.

In this study, we investigated whether the truncation of the CD63 tetraspanin for the creation of a new N-terminal is a successful approach that allows the incorporation of tags to facilitate EV immunoaffinity purification. Our aim was to understand if and how such changes in the tetraspanin may influence cellular EV production and CD63 localisation, continuing the work of Curley et al.^9^ who showed the effectiveness of this truncation. In this study, we selected to insert the novel, versatile peptide ALFA-tag on the N-terminal truncated CD63 to test this theory, and add a Nanoluciferase sequence on the C-terminus, that will allow their tracing via luminescence with high sensitivity. Firstly, we showed that the size of the EVs expressing ALFA-tCD63-Nluc or CD63-Nluc did not differ from control, unmodified EVs, but HEK293 cells produced fewer vesicles when transfected with ALFA-tCD63-Nluc. This was unexpected, as we and others have found that transfection for the expression of CD63 leads to an increased production of EVs compared to non-transfected cells^15^. Upon further investigation, our results showed that HEK293 cells produced adequate quantities of the ALFA-tCD63-Nluc protein that were comparable to the other CD63 constructs, but which was unable to properly localise on the EVs surface, as shown by the low luminescence of EV and the low CD63 levels in the ALFA-tCD63-Nluc EVs. Surprisingly, the ALFA-tCD63-Nluc construct was not eluted in the EV fraction, but in a later SEC fraction where soluble proteins are present. To further understand why the truncated CD63 was unable to localise on the EV surface after its cellular expression, we performed immunostaining on the transfected HEK293 cells and showed the abnormal spherical localisation of ALFA-tCD63-Nluc in the cells. These spherical formations were stained positive for the PROTEOSTAT^®^ dye, thus indicating their formation into aggresomes potentially leading to their degradation before being able to incorporate into the EV membrane.

This study aimed to highlight the necessity to adequately characterise EVs to ensure their integrity when modifying important elements that are known to be implicated in their biogenesis and biological function. CD63 is necessary for the biogenesis of EVs^19^ and attempts to identify the regions of the protein that are necessary for this function have demonstrated the importance of the large extracellular loop and the second, third, and fourth transmembrane domains^8,9^. Even though Curley et al., showed that the removal for the first 46 amino acids of CD63, which comprise the N-terminus, the first transmembrane domain, and part of the small extracellular loop led to the expression of the protein and its localisation on the EV surface with fluorescence microscopy, they did not quantify the magnitude of the truncated CD63 localisation^9^. Here we show that this same truncation, with the addition of the ALFA-tag and the Nluc sequences, not only impair the ability of HEK293 to produce more EVs, but also lead to the improper localisation of CD63 on the vesicular membrane.

At this point, it is worth acknowledging that others aimed to develop novel immunoaffinity capture methods by associating tags on CD63. For example, Rufino-Ramos et al., developed a method where a flag was inserted in the middle of the small extracellular loop of CD63 which allowed their immunoaffinity capture with flag-tag affinity beads^20^, but did not show how these changes affect the production of EV and CD63 localisation. Additionally, Bobbili et al., developed a method to isolate EV by incorporating a snorkel-tag on the C-terminus of CD81, and showed that this modification did not impair EV production compared to unmodified cells^21^.

Ultimately, we aimed to highlight how important it is to properly characterise EV both physically and biochemically, when utilising genetic engineering to modify them. This is to ensure the minimal effects of such changes in their morphology, biogenesis, and release, as it is unknown how these changes may also influence their therapeutic capacity.

## Electronic supplementary material

Below is the link to the electronic supplementary material.

Supplementary Material 1

## Data Availability

The data presented in this study is original and available upon request to the corresponding author.
