# Supplementary material for: A cautionary note on the potential pitfalls of using N-terminal truncated CD63 to label small extracellular vesicles
Source: Sci Rep. 2025 Mar 1;15:7261. doi: 10.1038/s41598-025-91597-6 (PMC11873209; doi:10.1038/s41598-025-91597-6)
Supplement: Supplementary file 1 — Supplementary Material 1 [file 41598_2025_91597_MOESM1_ESM.docx]

Supplementary material

**A cautionary note on the potential pitfalls of using N-terminal truncated CD63 to label small extracellular vesicles**

Elias Sulaiman, Derek M Yellon, Sean M Davidson*

The Hatter Cardiovascular Institute, University College London, 67 Chenies Mews, London WC1E 6HX, United Kingdom.

***Corresponding author.**

Professor Sean Davidson

The Hatter Cardiovascular Institute

University College London

67 Chenies Mews

London WC1E 6HX

Tel: +44 203 447 9894

email: [s.davidson@ucl.ac.uk](mailto:s.davidson@ucl.ac.uk)


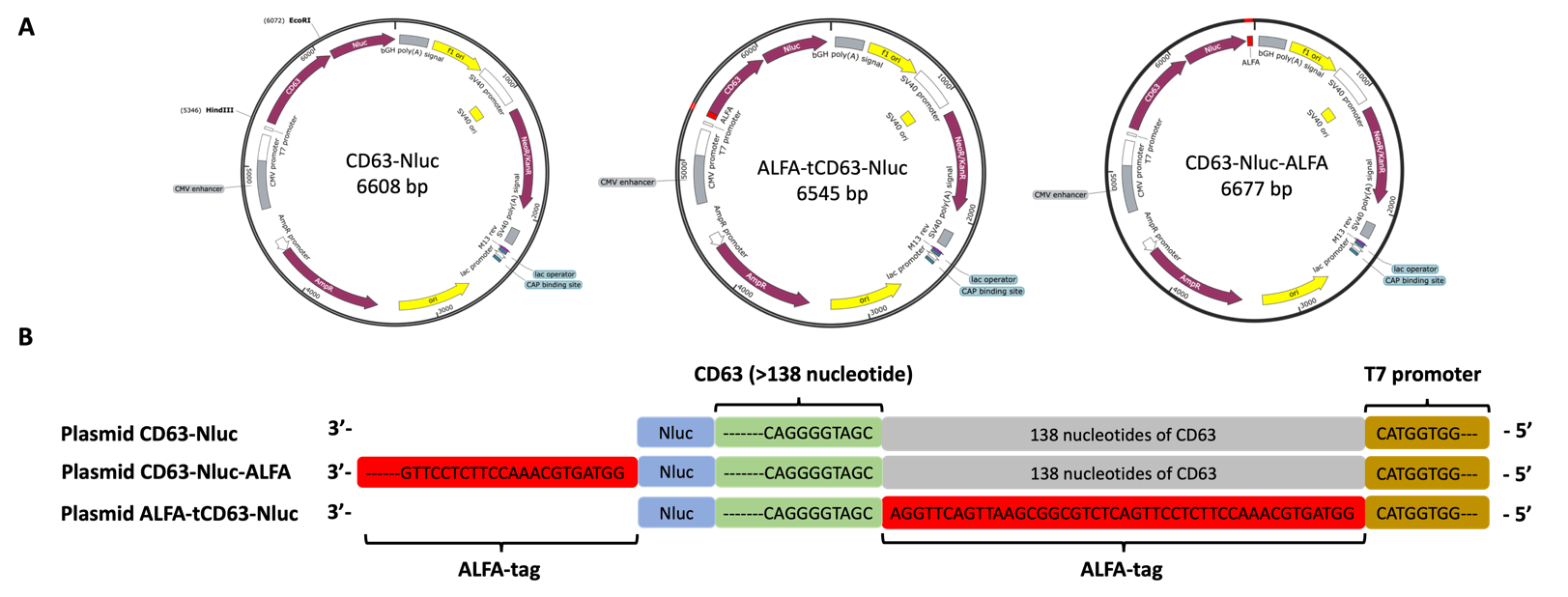


**Supplementary figure S1**: **Sequence analysis of the newly synthesised plasmid DNA encoding ALFA-tCD63-Nluc**. **(A)** Visual representation of the three plasmid DNA used in this study. In each plasmid, there is a CMV promoter before CD63 that allows CD63 transcription. Plasmids were visualised using SnapGene Viewer 6.2. **(B)** The ALFA-tCD63-Nluc plasmid was synthesised in our laboratory, and sent to Source Bioscience UK for sequencing. We aligned the sequences of all three plasmids (CD63-Nluc, CD63-Nluc-ALFA, and ALFA-tCD63-Nluc) to indicate the correct insertion of the ALFA tag and truncation of CD63. The plasmid fragments have the correct sequence for the T7 sequencing promoter that precedes the CD63 cDNA (brown colour). In the plasmid CD63-Nluc-ALFA, the CD63 cDNA remains unchanged, and the sequence for ALFA-tag (red) was inserted after the Nluc sequence (blue). For the ALFA-tCD63-Nluc, the first 138 nucleotides of CD63 were removed and the sequence for the ALFA-tag was inserted. The truncated CD63 begins from nucleotide 139 (green colour). Alignment of the sequences was performed with Jalview 2.11.4.1.

**Supplementary figure S2**: **Nanoparticle tracking analysis (NTA) of modified and un-modified sEV isolated from transfected HEK293 cells by ultrafiltration**. **(A)** Fraction 3.0 ml from size exclusion chromatography (SEC) is the most particle-rich from all EV isolations, regardless of the plasmid transfection that was induced. No
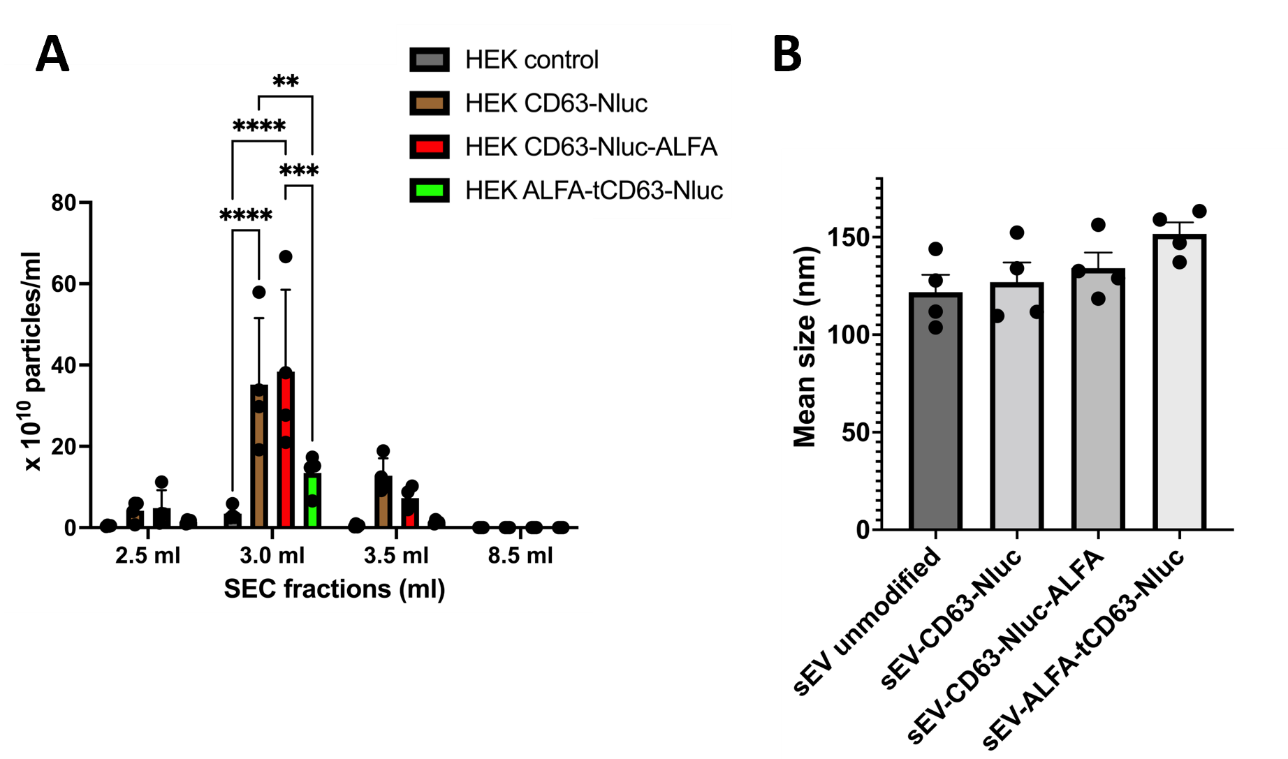
particles were detected in the late 8.5 ml fraction with NTA. **(B)** The mean size of EV from fraction 3.0 ml of each isolation, as measured with NTA. Data shown as mean± SEM (n=4), **p<0.01, ***p<0.001, & ****p<0.0001. HEK: human embryonic kidney cells 293; Nluc: nanoluciferase.


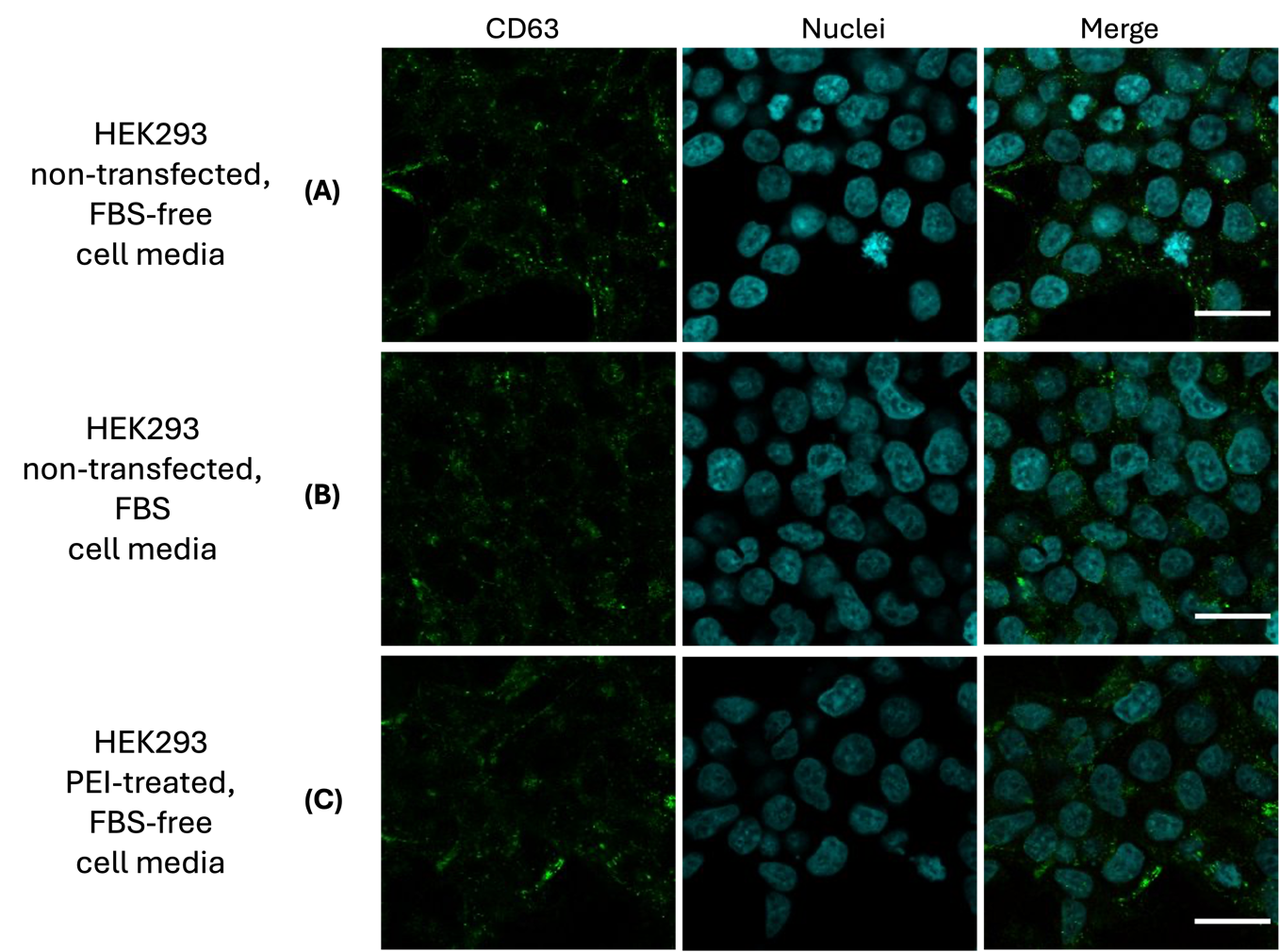


**Supplementary fig S3**: **Immunofluorescent staining of CD63 expression in HEK293 cells under different cell culture conditions**. Non-transfected HEK293 cells were stained for CD63 (green) and DNA (blue) and revealed no changes in CD63 expression when cultured in FBS-free **(A)** or FBS-containing **(B)** media, or when treated with PEI transfection reagent overnight in FBS-free media **(C)**. FBS: fetal bovine serum; HEK293: human embryonic kidney cells 293; PEI: polyethylenimine. Scale 30 um.


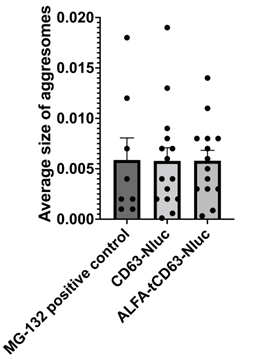


**Supplementary fig S4**: **Analysis of aggresome size of transfected HEK293 cells**. The immunofluorescence images were used for the quantification of the aggresome size in HEK293 cells expressing either the full length CD63-Nluc or the truncated ALFA-tCD63-Nluc protein. As a positive control, HEK293 cells were treated with the proteasome MG-132 inhibitor. Analysis was conducted with ImageJ. HEK: human embryonic kidney cells 293; Nluc: nanoluciferase.
